# Effectively Enhanced Photocatalytic Performance of BP/BiOBr 2D/2D Z-Scheme Heterojunction

**DOI:** 10.3390/molecules30030538

**Published:** 2025-01-24

**Authors:** Jian Feng, Xia Ran, Li Wang, Bo Xiao, Jinming Zhu, Zuoji Liu, Chaozhong Li, Rong Li, Guangwei Feng, Ke Xu

**Affiliations:** 1 School of Basic Medical Sciences, Guizhou Medical University, Guiyang 550025, China; jfeng@gmc.edu.cn (J.F.); ranxia@gmc.edu.cn (X.R.); mona19851228@126.com (L.W.); xiaobogzmu@163.com (B.X.); jinmingzhu@gmc.edu.cn (J.Z.); liuzuoji@gmc.edu.cn (Z.L.); lichaozhong@gmc.edu.cn (C.L.); lirong1@gmc.edu.cn (R.L.); fengguangwei@gmc.edu.cn (G.F.); 2 School of Chemistry and Materials Science, Guizhou Education University, 115 Gaoxin Road, Guiyang 550018, China

**Keywords:** BP/BiOBr, Z-scheme, hydrogen evolution, ciprofloxacin

## Abstract

Black phosphorus (BP) is a novel two-dimensional (2D) material with remarkable potential for use in environmental remediation and energy conversion. However, the practical application of BP is significantly limited by its low catalytic efficiency and poor structural stability. In this study, a Z-scheme BP/BiOBr 2D/2D heterojunction was fabricated using a simple solution reaction method at room temperature. The BP/BiOBr heterojunction exhibited significantly enhanced photocatalytic performance in the degradation of various organic pollutants and the production of hydrogen under visible light irradiation. This improved activity can be attributed to the efficient separation of photogenerated charges and the extended lifetime of charge carriers within the heterojunction. The durability and structural stability of the BiP-10 heterojunction were demonstrated through cycling tests, which maintained high photocatalytic efficiency over multiple uses. This study presents a promising approach to the development of BP-based photocatalytic materials for sustainable environmental and energy applications.

## 1. Introduction

Environmental pollution and the depletion of fossil fuels have emerged as significant challenges to the sustainable development of human society. These issues compel scientists to innovate efficient and eco-friendly technologies for harnessing renewable solar energy [1,2]. Photocatalysis, which employs semiconductor materials to catalyze chemical reactions under solar irradiation, has garnered considerable attention due to its potential applications in water splitting [3,4], the photodegradation of contaminants [5,6,7], and carbon dioxide reduction [8,9], among other areas. This technology is regarded as a promising solution to address energy and environmental challenges [10,11]. Photocatalysts typically exhibit a high recombination rate of photoinduced electrons and holes. Furthermore, the wide bandgap of photocatalysts limits their ability to harness visible light energy, which constitutes a significant portion of solar irradiance. These factors restrict the overall efficiency of photocatalysis. Narrowing the bandgap of semiconductors and constructing Z-scheme heterojunctions are considered effective strategies for enhancing photocatalytic efficiency [12,13].

For instance, g-C3N4 was employed to create g-C_3_N_4_/TiO_2_ heterojunctions, resulting in a significant enhancement in hydrogen production [14]. The interface structure and the direction of charge transfer confirmed its Z-scheme characteristics, which improved light absorption and charge separation, thereby enhancing photocatalytic efficiency. Numerous studies have demonstrated that the construction of Z-scheme heterojunctions mitigates low photocatalytic efficiency caused by charge recombination [15]. The energy alignment of the two semiconductors facilitates the efficient transfer of electrons and holes across the junction, effectively decoupling their recombination pathways. This spatial separation of charge carriers significantly extends their lifetime, allowing for more effective interactions with reactant molecules. Furthermore, the typical photogenerated electron transfer between the two components results in the retention of high-energy electrons and holes within a Z-scheme system, which increases the overall redox potential. Consequently, this system can drive more challenging and energetically demanding photocatalytic reactions, such as water splitting and CO_2_ reduction [16,17]. Therefore, selecting semiconductor materials with complementary bandgap energies is crucial for the successful implementation of Z-scheme heterojunctions.

Black phosphorus (BP) is an emerging two-dimensional (2D) material characterized by a layer-dependent bandgap, strong light absorption, and high carrier mobility [18]. Additionally, BP can be easily exfoliated into few-layer nanosheets, which can be efficiently integrated with other 2D materials or traditional semiconductors to create Z-scheme heterojunctions [19,20]. These advantages facilitate the application of high-performance BP-based heterojunctions in the field of photocatalysis. However, BP also has several inherent drawbacks when utilized as a photocatalyst. It is highly sensitive to oxygen and moisture, which can lead to rapid degradation and oxidation [21]. This instability significantly limits the practical applications and long-term durability of photocatalytic processes. Furthermore, illumination can induce photo-corrosion of BP, resulting in a reduction in photocatalytic activity over time. This presents a critical challenge for ensuring its sustainable use in photocatalysis. Various studies have demonstrated that the construction of BP-based heterojunctions can effectively mitigate BP oxidation and photo-corrosion [22,23]. More importantly, the combination of BP with other semiconductors can enhance the redox potentials of BP, leading to more effective and energetically favorable photocatalytic reactions. Bismuth oxybromide (BiOBr) is a layered semiconductor composed of [Bi_2_O_2_]^2+^ layers interleaved with double slabs of Br^−^ [24]. BiOBr has a bandgap of approximately 2.61–2.90 eV, making it suitable for absorbing visible light [25]. The surface area and morphology of BiOBr can be tailored during the synthesis process. Moreover, BiOBr exhibits excellent chemical stability under light irradiation and in various environmental conditions [26]. These features endow BiOBr with superior performance as a candidate for constructing heterojunctions with BP. In this study, we successfully fabricated a BP/BiOBr 2D/2D heterojunction, resulting in enhanced photocatalytic performance. The separation of photoinduced charges was assessed, and the Z-scheme mechanism was elucidated. This study provides a reliable reference for improving the stability and sustainability of BP through the construction of heterojunctions.

## 2. Materials and Methods

### 2.1. Preparation of BP/BiOBr Heterojunction

Bulk BP was synthesized using a phase transformation method as described in reference [27]. A specific amount of Sn, I_2_, and red phosphorus was vacuum sealed in a quartz tube. The sealed mixture was horizontally placed in a tube furnace and heated at a rate of 2 K/min to 923 K, where it was maintained for 5 h. The mixture was then cooled at a rate of 0.33 K/min to 773 K and held at this temperature for 2 h. Subsequently, the sample was allowed to cool naturally until it reached room temperature. The by-products were removed by ultrasonically treating the sample in toluene multiple times and refluxing for 15 min, until pure black phosphorus crystals were obtained.

BP nanosheets were synthesized as follows: 0.1 g of bulk BP was dispersed in 20 mL of anhydrous ethanol and then transferred into a 50 mL sealed stainless steel ball milling jar. Stainless steel balls with diameters of 10 mm and 5 mm, totaling approximately 100 g, with each type accounting for 50% of the total weight, were used for ball milling. The mixture was milled at a rate of 600 rpm for 6 h. The larger BP particles were removed by centrifugation at 5000 rpm, followed by further centrifugation at 12,000 rpm to separate the precipitate identified as small BP. Subsequently, 70 mg of small BP was added to 100 mL of *N*-methyl-2-pyrrolidone (NMP) and subjected to ice bath sonication (maintaining a temperature of 4 °C) for 8 h, resulting in a brown solution containing BP nanosheets.

BP/BiOBr heterojunction was prepared as follows: 10 mg of BP nanosheets were added to 20 mL of ethylene glycol and stirred for 30 min to form a homogeneous mixture. Subsequently, 0.485 g (1 mmol) of bismuth nitrate was incorporated and stirred until fully dissolved for 2 h, resulting in solution A. In a separate container, 0.151 g (1 mmol) of sodium bromate was dissolved in 20 mL of ultrapure water to obtain solution B. Solution B was then added to solution A and stirred for an additional 2 h to facilitate the reaction. The resulting white precipitate was collected via centrifugation and washed multiple times with ethanol and ultrapure water. The precipitate was then dried overnight at 50 °C to yield the sample BiP-10. By varying the amount of black BP nanosheets, a series of samples, including BiOBr, BiP-5, BiP-15, and BiP-20, were synthesized.

### 2.2. Characterization

X-ray diffraction patterns (XRD) were obtained using a Rigaku Smartlab diffractometer (Rigaku Corporation, Tokyo, Japan) with Cu Kα radiation (λ = 1.5406 Å) to analyze the phase structures of the samples at 40 kV and 40 mA. The recording range was 10° to 80°, with a resolution of 0.02° and a scan rate of 10° min^−1^. Transmission electron microscopy (TEM) and high-resolution TEM (HRTEM) images were obtained using a JEOL-2100F transmission electron microscope (JEOL, Tokyo, Japan) with an acceleration voltage of 200 kV to analyze the morphology and structure. The samples were ultrasonically dispersed in an ethanol solution, dropped onto a copper grid, and allowed to air-dry. Scanning electron microscopy (SEM) and elemental mapping images were captured using a JSM-4800F scanning electron microscope (JEOL, Tokyo, Japan). X-ray photoelectron spectroscopy (XPS) was conducted using a Thermo ESCALAB 250XI spectrometer (ThermoFisher, Waltham, MA, USA) with monochromatic Mg-Kα as the X-ray source (hν = 1486.6 eV) operating at 150 W to analyze the chemical states of the samples. Long-term vacuum pumping was employed to eliminate surface contaminants. The C1s peak at 284.8 eV was used as the reference to calibrate the binding energies. Electron spin resonance (ESR) spectra of radicals spin-trapped by 5,5-dimethyl-1-pyrroline N-oxide (DMPO) and 2,2,6,6-tetramethylpiperidine-1-oxyl (TEMPO) were recorded on a Bruker E500 spectrometer (Bruker, Karlsruhe, Germany) under visible-light irradiation. Photoluminescence (PL) and time-resolved PL spectra were detected using the Edinburgh FS5 fluorescence spectrometer at room temperature to analyze the optical properties of the samples. Electrochemical impedance spectroscopy (EIS) and transient photocurrent responses were conducted using a CH1660D electrochemical workstation (Chenhua, Shanghai, China) with a 300W Xe lamp as the visible light source. The catalyst attached to a cleaned glass substrate (ITO, 1 × 1 cm^2^) as the working electrode was prepared using the spin-coating method. A standard Si photodiode calibrated the light intensity. UV–Vis diffuse reflection spectroscopy (DRS) and UV–Vis spectra were taken at room temperature using a Shimadzu UV-2450 spectrophotometer (Shimadzu Corporation, Nagoya, Aichi, Japan) equipped with an integrating sphere and employing BaSO4 as a reference.

### 2.3. Photocatalytic Activity

The photocatalytic activity of the samples was assessed by measuring the degradation of ciprofloxacin (CIP, 10 μM), tetracycline (TE, 25 μM), oxytetracycline (OTC, 25 μM), levofloxacin (LVX, 25 μM), methylene blue (MB, 10 μM), and rhodamine B (RhB, 10 μM) in an aqueous solution under the irradiation of a 40 W LED. The illumination intensity used in the photocatalytic experiments was 35 mW/cm^2^. A total of 0.1 g of photocatalyst was added to 200 mL of the selected pollutant solution and stirred vigorously for 0.5 h. After reaching adsorption–desorption equilibrium, the white LED was turned on to initiate the photocatalytic degradation reaction. At specific time intervals, samples of the degradation solution were withdrawn and filtered using a 0.22 μm filter. The residual pollutant concentrations in the supernatants were analyzed using UV–Vis spectroscopy. The active species involved in the decomposition of CIP were investigated in the context of the BP/BiOBr heterojunction.

The photocatalytic hydrogen production was conducted in a Pyrex top-irradiation reaction vessel without the use of a co-catalyst. A catalyst weighing 50 mg was dispersed in 100.0 mL of distilled water, which was supplemented with 10 vol% triethanolamine as a sacrificial agent. The resulting mixture underwent degassing and was subsequently sealed. The concentration of hydrogen was measured using a microsensor multimeter (Unisense) equipped with Clark-type electrochemical microsensors for H_2_. The reaction was maintained at a temperature of 20 °C, utilizing a 300 W xenon lamp with a cut-off filter (λ ≥ 420 nm) as the visible light source.

## 3. Results and Discussion

**Figure 1:**
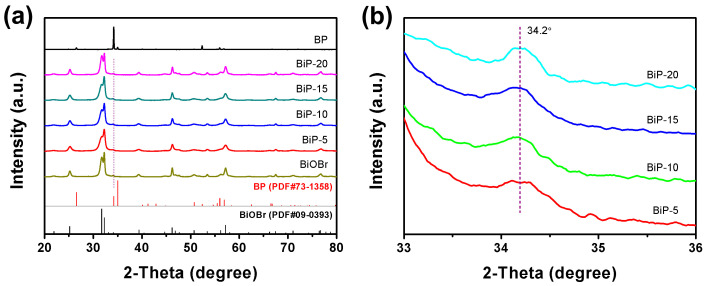
(**a**) XRD and (**b**) their corresponding partial magnifications for BP, BiOBr, and BiP heterojunctions.

The phase structures of BP, BiOBr, and BiP heterojunctions were analyzed using XRD. The XRD presented in Figure 1a demonstrate that both pristine BP and BiOBr exhibit high crystallinity, as evidenced by the close alignment of all diffraction lines with the standard reference patterns for BP (PDF#73-1358) and BiOBr (PDF#09-0393) [28,29]. The diffraction lines of BP observed at 34.2° and 35.0° correspond to the (040) and (111) crystal planes, respectively. Notably, the intensity of the (040) reflection is significantly greater than that of the (111) reflection. This disparity suggests the prominence of the {010} crystal planes, as documented in previous studies [30,31]. Such findings imply a potential preferential growth orientation of the {001} crystal planes of BP. The presence of diffraction lines from BiOBr in the BiP heterojunctions was anticipated. The observed broadening of the diffraction lines for BiOBr and BiPs indicates the presence of small crystallite sizes. However, only the diffraction peak of BP at 34.2° is detected in the BiPs samples, suggesting a low content of BP in the heterojunction. Importantly, the intensity of the diffraction peak at 34.2° gradually increases with the rising content of BP nanosheets (Figure 1b).

**Figure 2:**
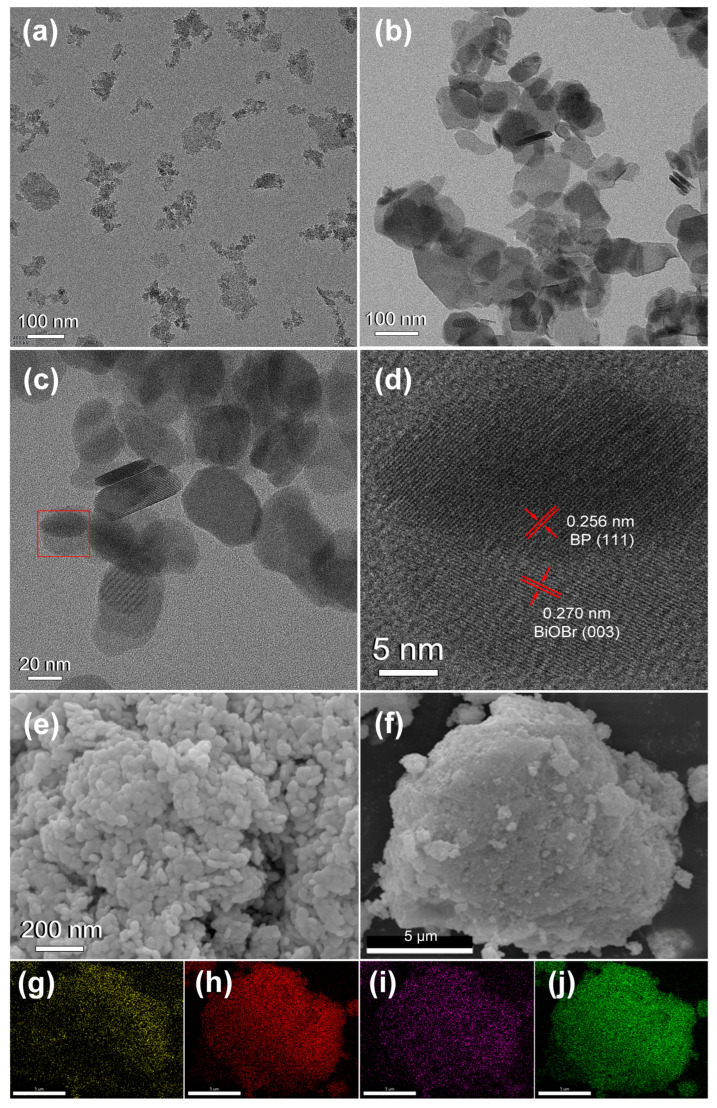
TEM images of (**a**) BP nanosheets, (**b**) BiOBr nanosheets, (**c**) BiP-10 heterojunction, and (**d**) HRTEM of BiP-10 heterojunction. SEM images of (**e**,**f**) BiP-10 heterojunction, and element mapping images of (**g**) O, (**h**) Br, (**i**) P, and (**j**) Bi.

The morphology and microstructural characteristics of the BP, BiOBr, and BiP-10 heterojunction were systematically investigated using TEM, HRTEM, SEM, and elemental mapping techniques. Figure 2a illustrates that BP exhibits a well-dispersed 2D sheet-like morphology. The lateral dimensions of BP nanosheets range from 53 to 127 nm, indicating a degree of heterogeneity. In contrast, BiOBr displays a relatively uniform layered structure (Figure 2b), with most BiOBr nanosheets having a lateral dimension of approximately 110 nm. BiOBr exhibits a smoother and cleaner morphology compared to BP nanosheets, which facilitates the attachment of BP nanosheets. The TEM (Figure 2c) and HRTEM (Figure 2d) images of the BiP-10 heterojunction reveal a dense arrangement of BP nanosheets on the surface of BiOBr nanosheets, forming a close 2D/2D contact. The interface between BP and BiOBr is clearly visible (Figure 2d), confirming the formation of the BiP heterojunction. The lattice spacings of 0.256 nm and 0.270 nm correspond to the (111) plane of BP and the (003) plane of BiOBr, respectively. The SEM image of the BiP-10 heterojunction (Figure 2e) shows an aggregated structure consisting of small sheets. The elemental composition and distribution within the BiP-10 heterojunction (Figure 2f) were analyzed using elemental mapping techniques (Figure 2g–j). The mapping images for O, Br, P, and Bi exhibit similar shapes, suggesting a uniform distribution of these elements. Collectively, these findings confirm the successful formation of the BiP-10 heterojunction.

**Figure 3:**
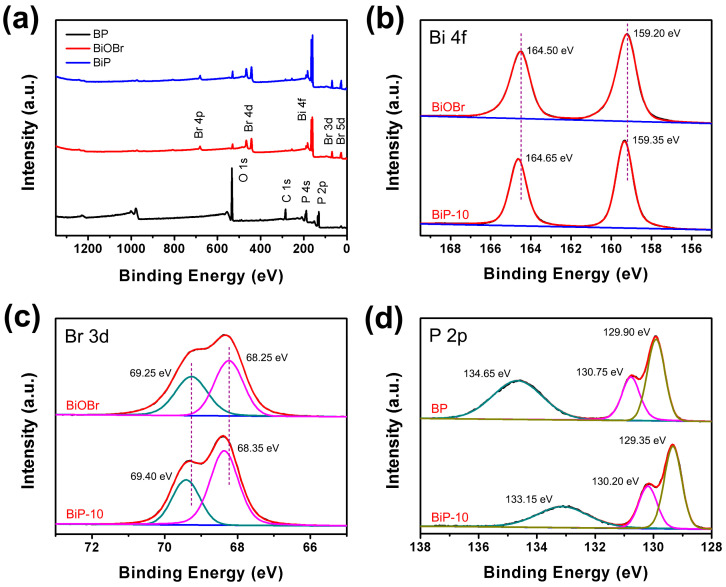
XPS spectra of BP, BiOBr, and BiP-10: (**a**) survey, (**b**) Bi 4f, (**c**) Br 3d, and (**d**) P 2p.

The chemical compositions of BP, BiOBr, and BiP-10 were analyzed using XPS. The XPS survey spectrum of BP, as illustrated in Figure 3a, indicates that the sample primarily contains P and O, with the oxygen arising from the surface oxidation of BP nanosheets. In contrast, BiP-10 predominantly comprises P, O, Bi, and Br, confirming its composition as a combination of BP and BiOBr. This finding validates the successful formation of a BP/BiOBr heterojunction. Notably, the peak intensity of oxygen significantly decreased in BiP-10, suggesting that the formation of heterojunctions effectively inhibits the surface oxidation of BP. Furthermore, the high-resolution Bi 4f spectra of the BiOBr and BiP-10 samples (Figure 3b) exhibit two distinct peaks corresponding to Bi 4f_5/2_ and Bi 4f_7/2_ [32]. Notably, these peaks shift from 159.20 eV and 164.50 eV in BiOBr to 159.35 eV and 164.65 eV in BiP-10, respectively. A similar phenomenon is observed in the Br 3d spectra of BiOBr and BiP-10 (Figure 3c). The two peaks corresponding to Br 3d_3/2_ and Br 3d_5/2_ in BiOBr, located at 68.25 eV and 69.25 eV, shift to 68.35 eV and 69.40 eV in BiP-10, respectively [33]. In the XPS spectra of P 2p in BP (Figure 3d), three distinct peaks at 129.90 eV, 130.75 eV, and 134.65 eV correspond to P 2p_3/2_, P 2p_1/2_, and oxidized phosphorus, respectively [34]. These peaks shift to 129.35 eV, 130.20 eV, and 133.15 eV in the BiP-10 heterojunction. Generally, variations in the binding energy of an element reflect changes in its electron density [35,36]. When an atom gains electrons, its binding energy decreases; conversely, the binding energy increases when an atom loses electrons. Therefore, the observed changes in the binding energies of Bi 4f, Br 3d, and P 2p suggest that electrons are transferred from BiOBr to BP in the BiP-10 heterojunction [37].

**Figure 4:**
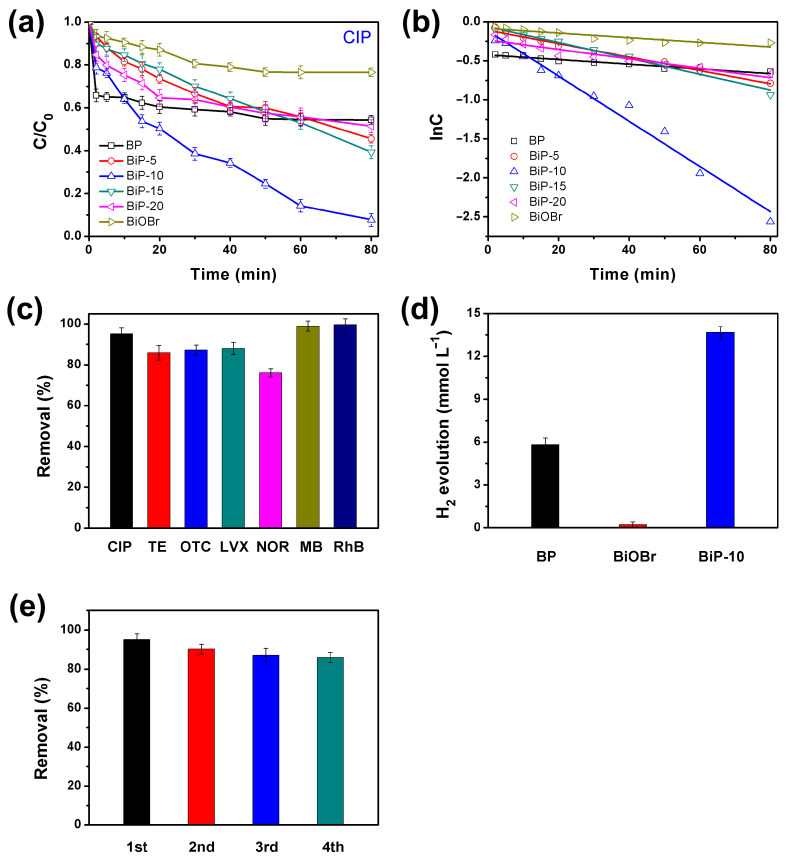
(**a**) Photocatalytic degradation of CIP over BP, BiOBr, and BiP heterojunction under 40 W white LED irradiation and (**b**) corresponding apparent rate constants. (**c**) Removal rate of different antibiotics and dyes over BiP-10. (**d**) Photocatalytic hydrogen evolution performance over BP, BiOBr, and BiP-10. (**e**) Recycling experiments of CIP degradation over BiP-10.

The photocatalytic activity of BP, BiOBr, and BiP heterojunctions was initially evaluated based on the degradation efficiency of CIP under 40 W white LED irradiation. The results are presented in Figure 4a. Pristine BP and BiOBr exhibited relatively low photocatalytic activity, degrading only 45.6% and 60.7% of CIP within 80 min, respectively. In contrast, the degradation efficiency of CIP significantly increased to 95.1% with the BiP-10 heterojunction. Notably, BiP-10 demonstrated optimal photocatalytic removal performance, enhancing the removal efficiency of CIP by approximately 49% and 34% compared to pristine BP and BiOBr, respectively. The corresponding kinetics plots of CIP degradation over BP, BiOBr, and BiP heterojunctions are presented in Figure 4b. The results indicate that the photocatalytic degradation of CIP follows a pseudo-first-order kinetics model [38]. The rate constants were determined to be 0.179 h^−1^ for BP, 0.52 h^−1^ for BiP-5, 1.74 h^−1^ for BiP-10, 0.61 h^−1^ for BiP-15, 0.36 h^−1^ for BiP-20, and 0.178 h^−1^ for BiOBr, respectively. Notably, the rate constant for BiP-10 is approximately 9.7 times greater than that of BP and BiOBr. Additionally, TE, OTC, LVX, NOR, MB, and RhB were selected to assess the multifunctional photocatalytic removal activity of BiP-10 for organic pollutants. The results are displayed in Figure 4c. The removal efficiencies for TE, OTC, LVX, NOR, MB, and RhB were approximately 85.9%, 87.2%, 88.0%, 76.0%, 98.9%, and 99.6%, respectively. This suggests that BiP-10 possesses a broad range of photocatalytic clearance capabilities.

The performance of visible light-driven photocatalytic H_2_ evolution was investigated to further confirm the multifunctional photocatalytic activity of BiP-10 without the use of a cocatalyst. Triethanolamine was employed as the sacrificial agent, and a 300 W Xe lamp (λ ≥ 420 nm) served as the visible light source. The H_2_ evolution rates were measured at 5.80, 0.12, and 13.67 mmol L^−1^ for BP, BiOBr, and BiP-10, respectively (Figure 4d). BiP-10 exhibited the highest rate of hydrogen evolution, which was approximately 2.3 times higher than that of BP and 114 times higher than that of BiOBr. Furthermore, the photocatalytic stability of catalysts is a critical aspect of their practical applications. Cycling experiments on the degradation of CIP over BiP-10 were conducted to assess its durability, as shown in Figure 4e. The used BiP-10 was recycled by air drying alone, without any additional treatments. The photocatalytic activity of BiP-10 demonstrated notable stability, achieving a degradation efficiency of 86% after four cycles. These results indicate that the formation of BiP heterojunctions can effectively enhance the photocatalytic stability of catalysts.

**Figure 5:**
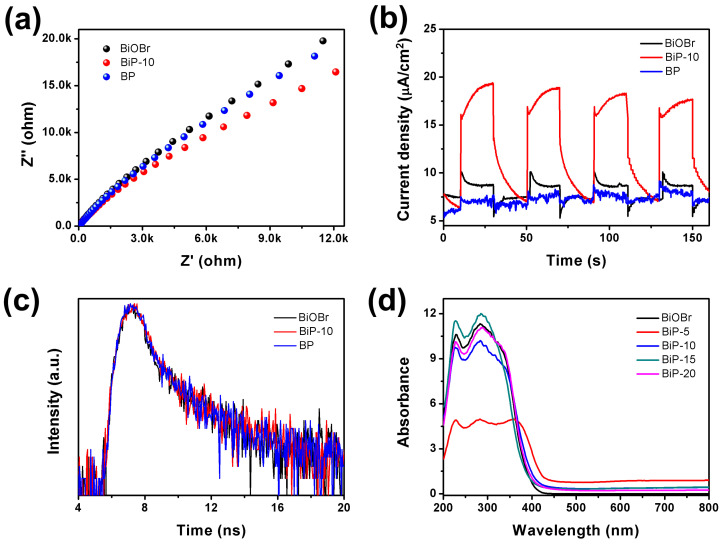
(**a**) EIS spectra, (**b**) transient photocurrent, (**c**) time-resolved PL spectra, and (**d**) UV–Vis DRS spectra of the samples.

The separation and transfer of photogenerated charges are critical factors influencing photocatalytic efficiency. The construction of BiP heterojunctions can significantly enhance this process, as demonstrated by the EIS Nyquist plots in Figure 5a. BiP-10 exhibited a smaller arc radius compared to BiOBr and BP, indicating lower interfacial resistance for charge transfer in BiP-10 [39]. Furthermore, the separation and migration of photogenerated charges in the samples were confirmed through transient photocurrent measurements (Figure 5b). The results indicate that BiP-10 displayed a superior photocurrent response compared to pristine BiOBr and BP, suggesting that BiP heterojunctions offer higher charge transfer efficiency [40]. Time-resolved photoluminescence (TRPL) spectra were conducted to analyze the lifetime of photogenerated charges in BiOBr, BP, and BiP-10 [41]. These decay spectra were fitted using a biexponential function. The average PL lifetimes for BiOBr, BP, and BiP-10 were 2.72 ns, 2.88 ns, and 3.11 ns, respectively. Notably, BiP-10 exhibited a longer lifetime compared to both BiOBr and BP, indicating an extended relaxation time for the photogenerated charges. The results from the EIS spectra, transient photocurrent measurements, and time-resolved PL spectra confirmed that the recombination of photogenerated charges in BiP-10 was effectively suppressed. This suggests that the formation of the BiP heterojunction facilitates charge separation and reduces the recombination of photogenerated charges, thereby enhancing photocatalytic activity.

UV–Vis DRS was utilized to evaluate the optical absorption characteristics of BiOBr and BiP heterojunctions, as illustrated in Figure 5d. The absorption edge of BiOBr was identified at approximately 423 nm, which corresponds to a band gap of 2.93 eV [42]. The UV–Vis DRS spectra revealed that the absorbance of the BiP heterojunction was significantly enhanced in the 400–800 nm range, indicating an improvement in the visible light utilization efficiency of the BiP heterojunction.

**Figure 6:**
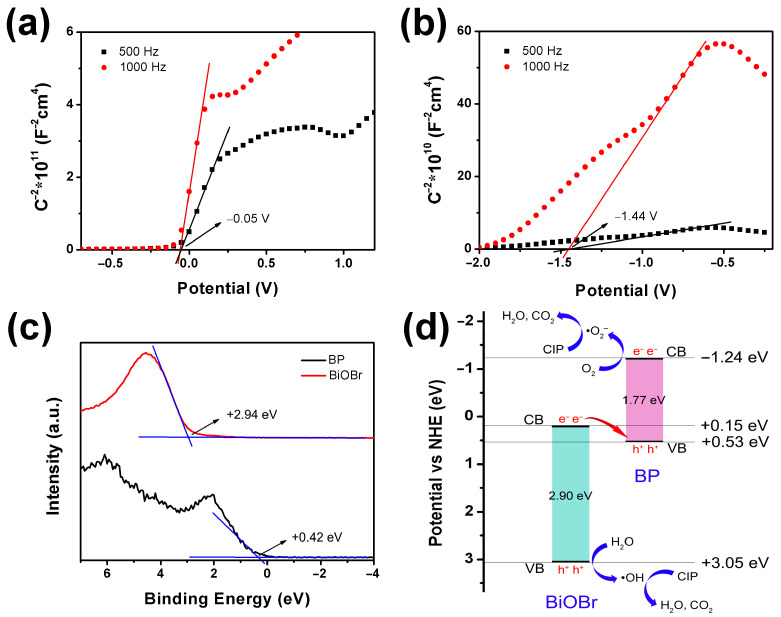
Mott–Schottky curves of (**a**) BiOBr and (**b**) BP, (**c**) XPS VB spectra, and (**d**) the band gap structures of BiOBr and BP.

The band structures of BiOBr and BP were determined using Mott–Schottky plots and VB-XPS spectra, which significantly influenced the photocatalytic activity of the catalysts. The flat band potentials of BiOBr and BP were assessed using Mott–Schottky curves. As depicted in Figure 6a,b, the flat band potentials were −0.05 eV and −1.44 eV relative to Ag/AgCl, respectively. The corresponding standard hydrogen electrode (NHE) potentials were calculated according to the method reported in the literature [43], yielding values of +0.15 eV and −1.24 eV, respectively. Notably, both BiOBr and BP exhibited positive slopes, indicating their characteristics as n-type semiconductors. Typically, the flat band potential of an n-type semiconductor is found to be close to the conduction band (CB) position. Therefore, the CB potentials of BiOBr and BP were approximately +0.15 eV and −1.24 eV relative to NHE, respectively. The valence band (VB) positions of BiOBr and BP were determined using XPS VB spectra, which were +2.94 eV and +0.42 eV, respectively. The respective VB potentials of BiOBr and BP (vs. NHE) were evaluated based on the reported method [44], resulting in +3.05 eV and +0.53 eV, respectively. The band gaps calculated from the CB and VB values for BiOBr and BP were 2.90 eV and 1.77 eV, respectively. The result for BiOBr was consistent with the band gap derived from UV–Vis DRS spectra, as illustrated in Figure 5d. Additionally, the band gap of 1.77 eV for BP indicates that BP possesses a thin-layer structure, which is attributed to the thickness-dependent band gap of BP [45,46]. The diagram illustrating the band structure of BiOBr and BP is presented in Figure 6d.

**Figure 7:**
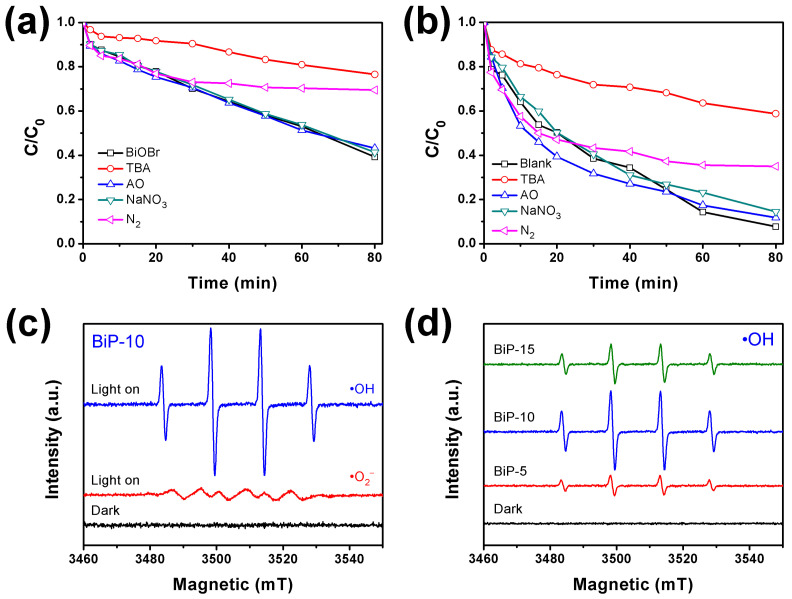
The impact of radical scavengers on the photocatalytic degradation of CIP using (**a**) BiOBr and (**b**) BiP-10 under 40 W LED irradiation, (**c**) the ESR spectra of DMPO-•O_2_^−^ and DMPO-•OH for BiP-10, and (**d**) the ESR spectra of DMPO-•OH with BiP-5, BiP-10, and BiP-15 under simulated solar light.

The scavenging experiments were conducted to identify the primary reactive species involved in the photocatalytic degradation of CIP over BiOBr (Figure 7a) and the BiP-10 heterojunction (Figure 7b). In this process, tert-butanol (TBA, 5 mL), ammonium oxalate (AO, 20 mg), NaNO_3_ (20 mg), and N_2_ (continuously bubbled) were employed as scavengers to capture •OH, h^+^, e^−^, and •O_2_^−^, respectively [47]. The results presented in Figure 7a demonstrate the degradation efficiencies of CIP on pristine BiOBr in the presence of these scavengers. The addition of TBA and N_2_ resulted in a significant reduction in CIP degradation efficiency, indicating the involvement of •OH and •O_2_^−^ in the photocatalytic degradation process. The CB potential of −0.47 eV and the VB potential of +2.43 eV for BiOBr exhibited sufficient capability to generate •O_2_^−^ and •OH. In contrast, in the BiP-10 photocatalytic system (Figure 7b), a marked decrease in CIP degradation efficiency was observed upon the addition of TBA and N_2_, suggesting that •OH and •O_2_^−^ play a crucial role in the degradation of CIP. Moreover, the photocatalytic degradation efficiency decreased even more significantly with the addition of TBA, indicating that •OH is more critical than •O_2_^−^ in this process.

To further confirm the dominance of •OH and •O_2_^−^ radicals in the photocatalytic degradation process, ESR spectra of DMPO-•O_2_^−^ and DMPO-•OH for BiP-10 were conducted and are presented in Figure 7c [48]. The ESR signals corresponding to •OH and •O_2_^−^ radicals were distinctly observed when subjected to simulated solar light irradiation. This observation indicates that •OH and •O_2_^−^ can be generated and subsequently contribute to the photocatalytic degradation process. The signal intensity of DMPO-•OH was significantly greater than that of DMPO-•O_2_^−^, suggesting a higher generation rate of •OH [49]. This finding is consistent with the scavenging experimental results for BiP-10. Additionally, ESR spectra of DMPO-•OH for BiP-5, BiP-10, and BiP-15 were conducted to investigate the changes in reactive species within the photocatalytic degradation systems. As illustrated in Figure 7d, the ESR signal intensity of DMPO-•OH in BiP-10 was comparatively greater than that in BiP-5 and BiP-15, indicating that BiP-10 can effectively convert H_2_O to •OH using photogenerated holes.

Based on the results from XPS, ESR spectra, and photocatalytic hydrogen evolution experiments, it can be concluded that the BiP heterojunction demonstrates the characteristics of a Z-scheme system [50,51]. The XPS analysis distinctly indicated that photogenerated electrons were transferred from BiOBr to BP. The ESR spectra revealed a considerable concentration of •O_2_^−^ and •OH radicals in the presence of the BiP-10 heterojunction. Conversely, if the charge transfer mechanism followed to the II-type mechanism within the heterojunction, the CB potential of BiOBr would be insufficiently negative to facilitate the reduction of O_2_ to produce •O_2_^−^ (O_2_/•O_2_^−^, −0.33 eV vs. NHE), while the VB potential of BP would be too negative to enable the oxidation of H_2_O (•OH/H_2_O, 2.27 eV vs. NHE) or OH^−^ (•OH/OH^−^, 1.99 eV vs. NHE) to generate •OH. Additionally, BiOBr exhibited low efficiency in photocatalytic hydrogen evolution. Consequently, the BiP heterojunctions display characteristics typical of Z-scheme systems. The photogenerated electrons preferentially migrate from the CB of BiOBr to the VB of BP, where they recombine with the photogenerated holes in the VB of BP. This process may facilitate the transfer of photogenerated charge carriers and inhibit their recombination in both BiOBr and BP. Furthermore, this arrangement of the energy band structure could maintain the high redox potential of BiOBr and BP, which is advantageous for the generation of active species during the photocatalytic degradation process [16,52]. As illustrated in Figure 6d, the photogenerated electrons and holes are retained in the CB of BP and the VB of BiOBr, respectively. The photogenerated holes in BiOBr oxidize H_2_O to generate •OH, while the photogenerated electrons in BP reduce O_2_ to •O_2_^−^. These reactions are thermodynamically feasible, as the standard redox potentials of •OH/H_2_O and O_2_/•O_2_^−^ are +2.27 eV and −0.33 eV, respectively [53,54]. Consequently, the BiP heterojunction demonstrates an enhanced rate of electron–hole separation and possesses strong redox capabilities, thereby improving the photocatalytic activity of the BiP heterojunction.

## 4. Conclusions

In summary, a 2D/2D BP/BiOBr heterojunction was successfully fabricated using a straightforward solution method at ambient temperature. The integration of BP with BiOBr resulted in a heterojunction that exhibits improved light absorption, enhanced charge separation, and a strong redox potential. This heterojunction effectively suppressed the recombination of photogenerated electron–hole pairs and demonstrated superior photocatalytic activity in the degradation of various organic pollutants and the evolution of hydrogen under visible light irradiation. The stability and durability of the BiP-10 heterojunction were confirmed through cycling experiments, which showed only a minor decrease in photocatalytic activity after multiple uses. The underlying mechanism was elucidated through various characterization techniques, revealing that the heterojunction operates on a Z-scheme system, where electrons are transferred from the CB of BiOBr to the VB of BP, leaving behind high-energy holes and electrons for efficient redox reactions. The primary reactive species identified in the photocatalytic process were •OH and •O_2_^−^ radicals, with •OH playing a more significant role. This study provides valuable insights into the design of efficient and stable BP-based photocatalytic systems for environmental remediation and energy production.

## Data Availability

The original data are available from the corresponding author upon reasonable request.
